# Nutrient transceptors physically interact with the yeast S6/protein kinase B homolog, Sch9, a TOR kinase target

**DOI:** 10.1042/BCJ20200722

**Published:** 2021-01-27

**Authors:** Zhiqiang Zhang, Ines Cottignie, Griet Van Zeebroeck, Johan M. Thevelein

**Affiliations:** 1 Laboratory of Molecular Cell Biology, Institute of Botany and Microbiology, KU Leuven, Leuven, Belgium; 2 Center for Microbiology, VIB, Kasteelpark Arenberg 31, B-3001 Leuven-Heverlee, Flanders, Belgium

**Keywords:** nutrient regulation, Protein Kinase B, S6 kinase, Sch9, transceptor, yeast

## Abstract

Multiple starvation-induced, high-affinity nutrient transporters in yeast function as receptors for activation of the protein kinase A (PKA) pathway upon re-addition of their substrate. We now show that these transceptors may play more extended roles in nutrient regulation. The Gap1 amino acid, Mep2 ammonium, Pho84 phosphate and Sul1 sulfate transceptors physically interact *in vitro* and *in vivo* with the PKA-related Sch9 protein kinase, the yeast homolog of mammalian S6 protein kinase and protein kinase B. Sch9 is a phosphorylation target of TOR and well known to affect nutrient-controlled cellular processes, such as growth rate. Mapping with peptide microarrays suggests specific interaction domains in Gap1 for Sch9 binding. Mutagenesis of the major domain affects the upstart of growth upon the addition of L-citrulline to nitrogen-starved cells to different extents but apparently does not affect *in vitro* binding. It also does not correlate with the drop in L-citrulline uptake capacity or transceptor activation of the PKA target trehalase by the Gap1 mutant forms. Our results reveal a nutrient transceptor–Sch9–TOR axis in which Sch9 accessibility for phosphorylation by TOR may be affected by nutrient transceptor–Sch9 interaction under conditions of nutrient starvation or other environmental challenges.

## Introduction

The ability of yeast cells to sense nutrients and respond appropriately to their availability is crucial for real-time adaptation and survival in changing environments and for competition with other organisms. Yeast cells have a wide array of nutrient-sensing systems for rapid adaptation to changes in the nutrient supply, including several types of plasma membrane nutrient sensors [1]. The latter include the glucose/sucrose-sensing G-protein coupled receptor (GPCR) Gpr1 [2] and the transporter-like, but transport-deficient glucose sensors, Snf3 and Rgt2 and amino acid sensor Ssy1 [3]. While much progress has been made in the elucidation of signaling pathways involved in the response to specific nutrients, much less is known about the nutrient-sensing systems involved in the regulation of cellular properties that are controlled by multiple essential nutrients, such as starvation response and proliferation rate [1,4]. We have previously identified several high-affinity transporters that are strongly induced upon starvation for their substrate and display an additional receptor capacity, functioning as transporter-receptors or ‘transceptors’. They function in short-term activation of the protein kinase A (PKA) pathway [5] in nutrient-starved cells upon re-addition of their missing substrate: Gap1 for amino acids [6–8], Mep2 for ammonium [9], Pho84 for phosphate [10–12], Sul1/Sul2 for sulfate [13], Ftr1 for iron and Zrt1 for zinc [14]. PKA plays a central role in nutrient regulation of yeast growth and growth-correlated stress tolerance and metabolic properties by controlling multiple transcription factors and metabolic enzymes [15]. The molecular mechanism by which the nutrient transceptors trigger activation of the PKA pathway, however, remains unclear. The transceptor category of nutrient sensors stands out in that they do not just regulate the transport and/or metabolism of the nutrient substrate, but have a wide-ranging effect on many cellular properties through their activation of PKA. Transceptors have also been identified in other organisms but they generally regulate the transport and/or metabolism of the nutrient substrate of the transceptor, at least as far as is known [16].

Sch9 is an AGC protein kinase family member, which is considered as the yeast ortholog of mammalian S6 kinase 1 (S6K). It is a direct phosphorylation substrate of the TOR (target of rapamycin) protein kinase and this phosphorylation is diminished under nutrient starvation and stress conditions as well as upon rapamycin inhibition of TOR [17]. Similar to PKA, TOR affects cell growth and related cellular and metabolic properties in response to multiple environmental conditions, including changes in nutrient supply [18]. Sch9 is required for TOR regulation of a major subset of these processes, in particular, coupling cell growth to division and thus controlling cell-size homeostasis [17,19]. Sch9 also affects yeast cell longevity [20,21], with its deletion causing a similar extension of longevity as calorie restriction [22,23]. In addition, Sch9 was shown to be required for PKA activation upon nitrogen re-addition to nitrogen-starved cells and was suggested to be a possible target of transceptor signaling [24]. The precise connection between Sch9 and upstream nutrient-sensing mechanisms, however, has remained unclear.

We now show that Sch9 physically binds both *in vitro* and *in vivo* with multiple nutrient transceptors. This indicates that nutrient transceptors can directly bind major intracellular regulatory proteins, just like classical receptors. Mutagenesis of putative Sch9-binding domains in Gap1, identified by peptide microarray mapping, affects Gap1-dependent re-upstart of growth with L-citrulline in nitrogen-starved cells to different extents, without any significant difference in transport activity between the mutant Gap1 proteins. Our results suggest that nutrient transceptor–Sch9 interaction may play a role in nutrient control of Sch9-dependent cellular processes.

## Materials and methods

### Yeast strains and plasmids

The *Saccharomyces cerevisiae* yeast strains used in this study are listed in Table 1, the plasmids used are listed in Table 2. The ZZ6–ZZ11 BiFC strains were constructed first in the BY4742 background by the transformation of PCR products and then mated with BY prototrophic strain JT26824. Following sporulation, single tetrads were selected to be completely prototrophic for the full genotype indicated in Table 1. The JT20867 *gap1Δ* strain was transformed with plasmids for the expression of HA-tagged wild type Gap1 and Gap1 mutants for GST pull-down and the re-upstart of growth. ZZ41–ZZ45 half-citrine single-tagged strains and ZZ47–ZZ48 double-tagged BiFC strains were constructed by transforming PCR products. ZZ46 strain was constructed by deleting *LEU2* in the ZZ6 strain for the transformation of the pTPQ128 and FBp709 plasmids.

**Table 1.** *S. cerevisiae* strains used in this study

Strain	Description	Reference
BY4742	*BY MATalpha his3 leu2 lys2 ura3*	[83]
JT20867	∑*1278b MATalpha ura3 gap1::KANMX*	Lab strain collection
JT26824	*BY prototrophic strain*	Lab strain collection
ZZ6	*BY MATalpha ura3 his3 SCH9-C-Citrine (SpHIS5) GAP1-N-Citrine (CaURA3)*	This study
ZZ7	*BY MATalpha his3 ura3 SCH9-C-Citrine (SpHIS5) MEP2-N-Citrine (CaURA3)*	This study
ZZ8	*BY MATalpha his3 ura3 SCH9-C-Citrine (SpHIS5) SUL1-N-Citrine (CaURA3)*	This study
ZZ9	*BY MATalpha his3 ura3 SCH9-C-Citrine (SpHIS5) PHO84-N-Citrine (CaURA3)*	This study
ZZ10	*BY MATalpha his3 ura3 TPK1-C-Citrine (SpHIS5) GAP1-N-Citrine (CaURA3)*	This study
ZZ11	*BY MATalpha his3 ura3 PKH1-C-Citrine (SpHIS5) GAP1-N-Citrine (CaURA3)*	This study
ZZ41	*BY MATa leu2 met15 ura3 his3 SCH9-C-Citrine (SpHIS5)*	This study
ZZ42	*BY MATalpha his3 leu2 lys2 ura3 GAP1-N-Citrine (CaURA3)*	This study
ZZ43	*BY MATalpha his3 leu2 lys2 ura3 MEP2-N-Citrine (CaURA3)*	This study
ZZ44	*BY MATalpha his3 leu2 lys2 ura3 SUL1-N-Citrine (CaURA3)*	This study
ZZ45	*BY MATalpha his3 leu2 lys2 ura3 PHO84-N-Citrine (CaURA3)*	This study
ZZ46	*BY MATalpha ura3 his3 leu2 SCH9-C-Citrine (SpHIS5) GAP1-N-Citrine (CaURA3)*	This study
ZZ47	*BY MATa leu2 met15 ura3 his3 SCH9-C-Citrine (SpHIS5) GNP1-N-Citrine (CaURA3)*	This study
ZZ48	*BY MATa leu2 met15 ura3 his3 SCH9-C-Citrine (SpHIS5) MUP3-N-Citrine (CaURA3)*	This study
ZZ49	*BY MATalpha ura3 his3 SCH9-C-Citrine (SpHIS5) GAP1-N-Citrine (CaURA3) NVJ1-RFP (KANMX)*	This study

**Table 2.** Plasmids used in this study

Plasmid	Description	Reference
pGEX-Sch9	GST-Sch9p expression	Bergsma J.
pGEX-4T-1-Pkh1	GST-Pkh1p expression	Lab plasmid collection
pGEX-4T-1	GST tag expression (N-terminus GST tagging)	GE Healthcare
pFL38-HA-Gap1	HA-Gap1 wild type expression	Lab plasmid collection
pFL38-HA-Gap1^FVF-DKD^	HA-Gap1^FVF-DKD^ expression	This study
pFL38-HA-Gap1^F231D^	HA-Gap1^F231D^ expression	This study
pFL38-HA-Gap1^V232K^	HA-Gap1^V232K^ expression	This study
pFL38-HA-Gap1^F233D^	HA-Gap1^F233D^ expression	This study
pFL38-HA-Gap1^S234E^	HA-Gap1^S234E^ expression	This study
pFL38	Empty expression plasmid	Lab plasmid collection
yN-URA3	pFA6a-split-yECitrine-CaURA3 (1–155 amino acids of citrine) N-half citrine protein tagging template	Geovani Lopez Ortiz, Mexico City, Mexico
yC-HIS5	pFA6a-split-yECitrine-SpHIS5 (155–238 amino acids of citrine) C-half citrine protein tagging template	Geovani Lopez Ortiz, Mexico City, Mexico
FBp709	Kar2_(1–135)_-mCherry-HDEL ER marker plasmid	[25]
pTPQ128	Sec7-DsRed Golgi marker plasmid	[26]
pFA6a-mRFP1-kanMX6	mRFP protein tagging template	[27]

Vector construction and cloning were performed in *Escherichia coli* DH5α strain (Invitrogen). Protein expression was performed by transforming pGEX plasmids into BL21*(DE3) strain (Invitrogen).

### Cultivation media and growth conditions

Yeast strains harboring plasmids were grown in SC-URA (0.17% yeast nitrogen base w/o amino acids w/o ammonium sulfate, 2% glucose, 0.077% CSM-URA, 0.5% ammonium sulfate) or YPD complete medium (2% (w/v) glucose, 2% (w/v) peptone, 1% (w/v) yeast extract) under continuous shaking at 200 rpm and at 30°C. For solid nutrient medium, 1.5% (w/v) Bacto agar was added. For nutrient starvation, cells were grown to exponential phase and subsequently transferred to the specific nutrient starvation medium. For nitrogen starvation, cells were incubated for 24 h in nitrogen-starvation medium (NSM) (0.17% w/v yeast nitrogen base w/o amino acids w/o ammonium sulfate, 4% glucose). For phosphate starvation, cells were incubated for 72 h in phosphate-starvation medium (0.57% yeast nitrogen base w/o phosphate, 0.079% CSM, 4% glucose). For sulfate starvation, the cells were incubated for 48 h in sulfate-starvation medium (4% glucose, 37 mM NH_4_Cl, 6.5 mM KH_2_PO_4_, 0.7 mM K_2_HPO_4_, 5 mM MgCl_2_, 1.7 mM NaCl, 0.9 mM CaCl_2_, 160 μM H_3_BO_4_, 0.6 μm KI, 0.7 μM ZnCl_2_, 0.7 μM CuCl_2_, 0.3 μM FeCl_3_, 0.4 mg/l calcium panthotenate, 0.4 mg/l thiamine HCl, 0.4 mg/l pyroxidine HCl, 2 mg/l biotin and the appropriate nucleotides and amino acids to complement auxotrophic markers). The starvation media were refreshed every 24 h.

*Escherichia coli* strains were grown at 37°C in Luria Broth (LB) medium containing 0.5% (w/v) yeast extract, 1% (w/v) Bacto tryptone, and 1% (w/v) sodium chloride (pH7.5). For solid nutrient medium, 1.5% (w/v) Bacto agar was added. Selection of transformants was performed in the presence of 100 μg/ml ampicillin.

### Re-upstart of growth in nutrient-starved cells

Cells were pre-cultured in 3 ml of SC-URA medium with overnight shaking. The next day, the cells were diluted to OD_600nm_ 0.3 and grown for ∼4 h to exponential phase. Subsequently, the cells were collected by centrifugation and resuspended in NSM for 24 h in a shaking incubator. For determination of growth curves, cells were inoculated to OD_600nm_ 0.15 in a 96-well plate with either NSM containing 1 mM L-citrulline or SC-URA medium. The cultures were grown at 30°C for the indicated time and OD_600nm_ was measured every h in a Multiscan GO Microplate Spectrophotometer (Thermo Scientific).

### Transport assay with radioactively labeled L-citrulline

Cells were pre-cultured and starved for nitrogen for 24 h as described above. Cultures were centrifuged at 3000 rpm for 5 min to collect cell pellets, and washed once with 1 ml nitrogen-starvation medium. Then the cells were resuspended in fresh nitrogen-starvation medium to a concentration of 200 mg wet weight/ml. Forty microliters of cell suspension was added to a glass test tube and incubated in a 30°C water bath for 15 min. Ten microliters of 5 mM L-citrulline (mixed by unlabeled and [^3^H] labeled L-citrulline (ViTrax) to obtain ∼2000 ± 500 cpm.nmol^−1^) was added to the cells and incubated in a 30°C water bath for 1 min, before adding 5 ml ice-cold water to stop the reaction. Cells were then collected by filtration through a glass microfiber filter (Whatman GF/C, retention particle size 1.2 mm) prewet with 4 ml of 50 mM L-citrulline solution, and immediately washed 2 times with 5 ml of ice-cold water. Then the filter with the cells was transferred to a scintillation tube with 4 ml Ultima-Flo M scintillation fluid (PerkinElmer). Ten microliters of 5 mM radioactive L-citrulline solution was directly added to a scintillation tube for specific radioactivity measurement. The radioactivity was counted in a scintillation counter (Beckman Coulter LS6500), and transport activity is expressed as nmol L-citrulline.min^−1^.

### Construction of BiFC strains

A strain constructed for the determination of protein–protein interaction by BiFC assay is referred to as ‘BiFC strain’ in this paper. The endogeneous genes in these BiFC strains were C-terminally tagged with the Citrine fluorescent protein C-terminal or N-terminal half, which was achieved by transformation of tail PCR products using Q5 polymerase (NEB). Flanking regions of 50 bp homologous to the tagged gene were added to the Citrine N-terminal or C-terminal half, using the template plasmids yN-URA3 and yC-HIS5, that contain the *CaURA3* or *SpHIS5* marker, respectively, for selection. The resulting PCR constructs were transformed into the yeast cells for genomic integration. Correct insertion was confirmed using ExTaq Polymerase with primers outside of the inserted cassette on genomic DNA obtained with phenol-chloroform extraction and being sequenced. The Nvj1-RFP tagging was done similarly by the transformation of PCR product with 50 bp homologous to Nvj1, using template plasmid pFA6a-mRFP1-kanMX6 containing a KanMX marker cassette.

### Plasmid construction

Mutations were inserted in the pFL38-HA-Gap1 plasmid, using PCR, digestion and ligation. Oligos containing the desired Gap1 mutations were ordered from IDT (Integrated DNA Technologies). PCRs were conducted using Q5 (NEB) or ExTaq polymerase (Takara Clontech). Both plasmids and PCR amplicons were digested with *Kpn*I and *Age*I restriction enzymes (NEB). Plasmids were constructed using standard molecular biology methods. PCR and restriction digestion products were purified with Gel Wizard purification kit (Promega). Ligation was achieved with T4 DNA Ligase (NEB) followed by transformation into *E. coli* strain DH5α for amplification. The resulting plasmids were isolated and sequenced by the VIB genetic service facility using the applied biosystems 3730XL DNA analyzer (ABI) and products provided by the manufacturer.

### Confocal microscopy

To evaluate the *in vivo* interaction in the constructed BiFC strains, 3–5μl of appropriately starved cell culture was spotted on glass slides and immediately examined under the microscope. All images were acquired using an Olympus IX81 FluoviewTM FV1000 microscopy system equipped with UPLSAPO 60x O NA 1.35 objective lens, running FV10-ASW software. Images were analyzed using Fiji (ImageJ). The citrine BiFC signal was observed with a 488 nm laser for excitation, paired with a 530/30 nm filter. The Sec7-DsRed and the Nvj1-RFP were observed with a 561 nm laser for excitation, paired with a 582/15 nm filter. Sequential steps were taken for colocalization of multiple fluorescently tagged proteins to avoid unspecific excitation.

### DAPI and FM4-64 staining

For DAPI staining, 5 mg/ml DAPI (Sigma–Aldrich) stock solution was made in DMSO (Sigma–Aldrich). A final concentration of 5 µg/ml was added to the 24 h nitrogen-starved cell culture which was shaken at 30°C for 30 min. The cells were observed with a confocal microscope using a DAPI filter. For FM4-64 vacuolar membrane staining according to Pan et al. [28], 5 mg/ml FM4-64 (Invitrogen) stock solution was made in DMSO (Sigma–Aldrich). A final concentration of 5 µg/ml was added to the 24 h nitrogen-starved cell culture, which was then shaken at 30°C for 30 min in the dark. The cells were spun down and washed with fresh NSM. The pellet was resuspended in NSM and incubated at 30°C for 2 h. The cells were observed with a confocal microscope using an RFP filter.

### GST pull-down

#### *E. coli* protein expression

The *E. coli* BL21 Star (DE3) strain (Invitrogen) was transformed with the pGEX-4T-1, pGEX-4T-1-Sch9 and pGEX-4T-1-Pkh1 plasmids according to standard transformation protocols. Transformant colonies were picked into 3 ml overnight pre-culture in LB-ampicillin. The whole pre-culture was used to inoculate 500 ml of LB-ampicillin. The cells were grown at 37°C till OD_600nm_ of about 0.8. Protein expression was induced by adding 0.8 mM IPTG and the cells were incubated at 18°C with 200 rpm overnight shaking. Cell pellets were harvested by centrifugation, washed with ice-cold PBS, flash frozen in liquid nitrogen and stored at −80°C till further processing.

#### Cell lysis and preparation of GST-tagged protein bound to beads

For protein extraction, the cells were resuspended in *E. coli* lysis buffer (1x PBS, 0.8% Triton X-100, 2 mM MgCl_2_, 2 mM DTT, 0.2 mg/ml lysozyme) and lysed by 3 times 20 s sonication. The lysate was then spun down at 4°C 14 000 rpm for 15 min to remove cell debris. The supernatant was transferred into a fresh Eppendorf tube and Glutathione sepharose beads (GE Healthcare) solution (1 : 1 beads and lysis buffer) was added to the lysate for binding of the GST-tagged protein. The Eppendorf tubes were inverted on a roller drum for 2 h in the cold room. The beads were then collected by centrifugation at 2000rpm for 2 min and washed 3 times with wash buffer (1x PBS, 0.4% Triton X-100, 2 mM MgCl_2_, 2 mM DTT). Beads bound with GST-tagged protein were ready for the pull-down.

#### Yeast protein expression and cell lysis

A yeast strain expressing HA-tagged Gap1 from a plasmid was starved for nitrogen for 24 h. After starvation, the cells were pelleted by centrifugation, washed with ice-cold PBS, flash frozen in liquid nitrogen and stored frozen at −80°C till extraction. For extraction, ice-cold yeast lysis buffer (1x PBS, 0.2% Triton X-100, 10% glycerol, 2.5 mM MgCl_2_, 1 mM DTT, 10 mM NaF, 0.4 mM Na_3_VO_4_, 0.1 mM beta-glycerophosphate, protease inhibitor mix EDTA-free by Roche, 1 mM PMSF) and glass beads were added to the pellets. The cells were broken by vortexing 3 times for 20 s in a fast prep machine. The extracts were spun down at 14 000 rpm for 15 min to remove cell debris. The supernatant was transferred into a fresh Eppendorf tube. Glutathione sepharose beads (GE Healthcare) solution (1 : 1 beads and lysis buffer) was added to the lysate to remove unspecific binding proteins, inverting on a roller drum for 2 h in the cold room. The supernatant with HA-Gap1 was then separated from the beads by centrifugation at 2000 rpm and used for the pull-down assay. Fifty microliters of the supernatant was kept separately as ‘Input’ to detect HA-Gap1 expression with Western blot.

#### Pull-down assays

The *E. coli* beads bound with GST-tagged protein and yeast lysate with HA-Gap1, prepared as described above, were mixed and incubated on the roller drum for 2 h in the cold room to allow for protein–protein interaction. The beads were then collected by centrifugation and washed 3 times with PBS-T. The liquid was completely removed from the beads by pipetting with a syringe. Finally, sampling buffer was added to the beads and heated for 10 min at 65°C after which the resulting medium was loaded on an SDS–PAGE gel.

### Western blot

Ten microliters of input or pull-down sample was run on NuPAGE® Bis-Tris Mini Gel. The gels were run in NuPAGE® MOPS SDS Running buffer at 150 V for ∼1.5 h. The proteins were then transferred to a nitrocellulose membrane (HybondC extra, GE Healthcare) by blotting using 400 mA for 70 min in blotting buffer (NuPAGE® MOPS SDS Running buffer, 20% v/v methanol). The membranes were then blocked with 5% non-fat milk at room temperature for 2 h. After blocking, the membranes were incubated overnight in 2.5% non-fat milk with anti-HA-peroxidase antibody (3F10, Roche) 1 : 1500 diluted, at 4°C in a cold room. The next day, the membranes were washed 2 times for 10 min with PBS-T before being layered with Western Bright TMQuantum and Western Bright TM Peroxide for visualization in a LAS 400 mini machine. All original Western blots are shown in Supplementary document S1.

#### Quantification of Western blot bands

Western blot bands were quantified using Fiji (ImageJ) [29]. Calculations for comparing the pull-down bands intensity were done as follows. First, the band intensity from the membrane of pull-downs was divided by the band intensity from the membrane of ‘Input’, to normalize for the expression level, as the Gap1 mutant forms showed different expression levels. Subsequently, the normalized intensity of the mutant forms was calculated as percentage of the wild type intensity.

### Mapping with peptide microarrays and substitution scan

#### Preparation of the PEPperCHIP® peptide microarrays

For the peptide screening, a Gap1 peptide microarray was produced with each peptide 15 amino acids in length and 14 amino acids overlap. A total of 602 different peptides were synthesized on the microarray in duplicate (1204 spots in total) to cover the complete Gap1 protein. The Gap1 peptide map can be found in Supplementary document S2. For the substitution scan of motif ‘GVKGYGEAEFVFSFI’, amino acid residues in each position of this motif were replaced by all 20 protein amino acids. In total, 300 different peptides (15 × 20) were synthesized in triplicate (900 spots in total). The substitution peptide map can be found in Supplementary document S3.

#### Mapping procedure

For both the peptide screening and substitution scan, first pre-staining of the PEPperCHIP® Peptide Microarray was done with the secondary antibody rabbit anti-GST Dylight680 (1 : 2000) to determine possible background interactions. Then, 50 µg/ml, 100 µg/ml or 500 µg/ml GST-Sch9 or the same amount of GST as negative control were incubated with the microarray in incubation buffer (washing buffer with 10% MB-070 blocking buffer from Rockland) for 16 h at 4°C and shaking at 140 rpm. The microarray was then washed for 2 × 10 s with washing buffer (PBS, pH 7.4 with 0.005% Tween 20). Rabbit anti-GST Dylight680 (1 : 2000) antibody was used to detect GST-Sch9, after 2 h staining in incubation buffer at room temperature. HA control peptides were subsequently stained as internal quality control to confirm the assay quality and peptide microarray integrity. Finally, the microarray was scanned with a LI-COR Odyssey Imaging System and microarray image analysis was performed with PepSlide® Analyzer.

### Measurement of trehalase activity

Trehalase activation was determined as previously described [30] with minor adaptations. Five-hundred milliliters of a 24 h nitrogen-starved cell culture was incubated on ice for 30 min, and then collected by centrifugation for 10 min at 3000 rpm. The cells were then resuspended, washed with nitrogen-starvation medium and transferred to 50 ml falcon tubes. The pellets were then resuspended in nitrogen-starvation medium with a final concentration of 25 mg wet weight/ml. Twenty-six milliliters resuspended cell culture was then shaken in a 30°C water bath for 30 min. L-citrulline was added at time point 0. Samples of 50 mg cells were taken at different time points, and directly added to 40 ml of ice-cold water to stop the reaction. Cells were then collected by centrifugation and resuspended in 500μl ice-cold extraction buffer (100 mM MES buffer pH7.0, 50 μM CaCl_2_). The suspension was then transferred to a screw cap tube with glass beads, and lysis was achieved with 2 times fastprep shaking. After centrifugation for 15 min at 14 000 rpm, 150 μl of supernatant was loaded on a dialysis system containing dialysis buffer (10 mM MES buffer pH7.0, 100 μM CaCl_2_), for overnight removal of glucose. One-hundred microliters of the lysate was transferred from the dialysis system to a 96-well plate, for determination of trehalase activity and protein concentration.

The amount of glucose liberated after 30 min incubation of 10 μl of the extract with trehalase buffer (250 mM trehalose, 25 mM MES pH7.0, 50 μM CaCl_2_) was determined using the glucose oxidase-peroxidase method by addition of GOD-PAP reagent (Dialab). After 15 min incubation at 30°C, the absorbance at 505 nm was measured, indicating the concentration of glucose. The protein concentration was also determined, with the standard Lowry method [31]. The specific trehalase activity was expressed as nmol glucose.min^−1^.(mg protein)^−1^.

### Availability of data and materials

All data are included within the main article and its Supplementary information. All constructs and yeast strains are freely available upon request.

## Results

### Nutrient transceptors and Sch9 interact *in vivo*

**Figure 1.**
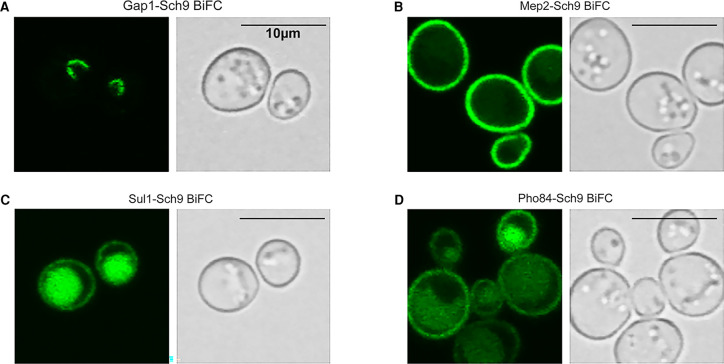
*In vivo* BiFC assay of the interaction between the nutrient transceptors Gap1. Mep2, Sul1 or Pho84 and the Sch9 protein kinase. *In vivo* interaction between Sch9 and Gap1, Mep2, Sul1 or Pho84 was tested. Strains were constructed for the BiFC assay by tagging the transceptors with N-half of citrine fluorescent protein and Sch9 kinase with C-half of citrine. After substrate-specific starvation (see Materials and methods), samples were taken directly from the culture and imaged with a confocal microscope. Images of BiFC citrine fluorescent signal (left) and the DIC channel (right) are shown. (**A**) The Gap1–Sch9 BiFC strain showed in each cell a cytosolic stripe or arc, which was later found to locate at the nucleus–vacuole junction. (**B**) The Mep2–Sch9 BiFC strain showed a bright signal localized at the plasma membrane in each cell. (**C**) The Sul1–Sch9 BiFC strain showed a weak signal at the plasma membrane and a stronger signal in the vacuole. (**D**) The Pho84–Sch9 BiFC strain showed a similar but stronger plasma membrane signal and a weaker signal in the vacuole. All the scale bars indicate 10 µm.

We have used a Bimolecular fluorescence complementation (BiFC) assay [32,33] to examine the *in vivo* interaction of Sch9 with the nutrient transceptors Gap1, Mep2, Sul1 and Pho84, respectively. The BiFC strains were constructed by fusing intragenomically the C-terminus half of the gene encoding the Citrine fluorescence protein [34] to the C-terminus of Sch9 and the N-terminus half to the C-terminus of the nutrient transceptors. The intragenomic fusion was achieved by transforming an appropriate PCR product having flanking sequences homologous to the end of the genes of interest into the target strain [35]. No BiFC signal could be observed in the cells during exponential growth (Supplementary Figure S1A), because of the repression of nutrient transceptor expression in the complete growth medium. To induce high expression of the respective nutrient transceptors, the cells were first grown to exponential phase after which they were starved for the substrate of the transceptor, as previously described [8,9,11,13]. Fluorescence signals were then detected with confocal fluorescence microscopy. The results show that all four nutrient transceptors generated clear fluorescence signals in cells of the respective BiFC strains (Figure 1). This indicates that all these nutrient transceptors likely bind with Sch9 *in vivo* and that the binding is especially prominent when the expression of the transceptors is strongly enhanced upon starvation for their substrate. It is well known that the binding of the citrine halves is irreversible once the two interacting partner proteins have brought the two halves together to restore the complete fluorescent citrine protein [36]. Hence, the binding strength cannot be evaluated from the strength of the BiFC signals. Nonetheless, the intracellular localization of the BiFC signal was different for the four nutrient transceptors (Figure 1). The Gap1–Sch9 BiFC signal was only visible in the cytosol as a stripe or arc (Figure 1A), although Gap1 has a major plasma membrane localization in nitrogen-starved cells [37]. For the ammonium transceptor, Mep2, on the other hand, nearly all of the BiFC signal was present at the plasma membrane, which is also the expected location of Mep2 [38] (Figure 1B). For the sulfate transceptor, Sul1, weak staining was visible at the plasma membrane, the expected location, and strong staining in the vacuole probably due to endocytosis and vacuolar targeting of the Sul1–citrine–Sch9 complex (Figure 1C). For the phosphate transceptor, Pho84, a similar pattern was observed as for Sul1, except that in most cells, plasma membrane staining of the Pho84–citrine–Sch9 complex was more pronounced and vacuolar staining less pronounced (Figure 1D). As negative controls for the observed BiFC interactions, the half-citrine single-tagged strains (strains ZZ41–ZZ45) were tested under the same conditions and no fluorescent signal was observed in all of them (Supplementary Figure S1B).

To identify the location of the cytosolic Gap1–Sch9 BiFC signal, DAPI staining, FM4-64 staining and expression of marker proteins in different organelles were performed (Supplementary Figure S2). The BiFC signal was always attached to the nucleus, partially colocalizing with the perinuclear ER and the vacuolar membrane. As the Gap1–Sch9 BiFC signal looked very similar to the localization of the nucleus–vacuole (NV) junction protein Nvj1 tagged with GFP [28], their colocalization was tested by tagging Nvj1 with RFP in the Gap1–Sch9 BiFC strain. Nvj1-RFP showed perfect colocalization with the BiFC signal, which indicated that the location of the BiFC signal is at the NV junction (Supplementary Figure S2). The NV junction is considered as the site for lipid droplet synthesis [39] and is essential for piecemeal microautophagy of the nucleus (PMN) under stress conditions like nutrient starvation [40,41]. Our hypothesis on the localization of the Gap1–Sch9 interaction is described below in the discussion. We also measured uptake of 1 mM L-citrulline, which is only taken up by Gap1 in yeast, in the cells from the BiFC experiments expressing the Gap1 and Sch9 half-citrine fusion proteins (Supplementary Figure S3A). L-citrulline uptake was only slightly, but significantly, reduced indicating that adequate levels of active Gap1 were still present in the plasma membrane. This indicates that there is an excess of Gap1 in the cell compared with Sch9, since the intracellularly located complexed Gap1 is not responsible for the uptake of L-citrulline.

### Sch9 and Gap1 interact *in vitro*

**Figure 2.**
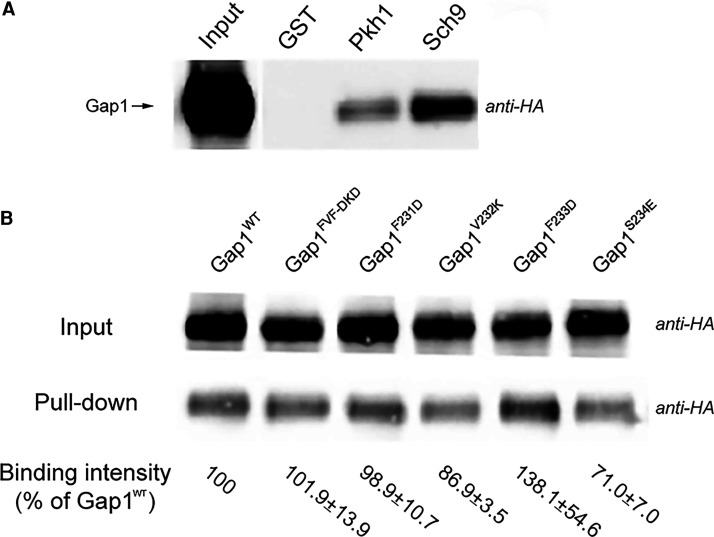
Detection of *in vitro* interaction using a GST pull-down assay. (**A**) HA-tagged wild type Gap1 was pulled down by GST-tagged Pkh1 or Sch9, and detected with anti-HA-peroxidase antibody. *Lane 1*: input from yeast cell extract directly loaded on the gel indicating the expression level of HA-Gap1 after 24 h nitrogen starvation. *Lane 2*: negative control with only GST tag showing that no HA-Gap1 got pulled down unspecifically. *Lane 3*: HA-Gap1 pulled down by GST-Pkh1. *Lane 4*: HA-Gap1 pulled down by GST-Sch9. (**B**) HA-Gap1 mutant forms pulled down by GST-Sch9. *Upper row input 1-6*: extract of yeast cells expressing HA-tagged Gap1^WT^, Gap1^FVF-DKD^, Gap1^F231D^, Gap1^V232K^, Gap1^F233D^ and Gap1^S234E^ was directly loaded on the gel for determination of their expression level with anti-HA-peroxidase antibody. *Lower row pull-down 1–6*: HA-tagged Gap1^WT^ or mutant forms were pulled down by GST-Sch9. The quantification of band intensity below indicates the signal strength as a percentage of the signal with Gap1^WT^, corrected with the expression level of the input and calculated as a mean of two independent experiments ± SD. There is no significant difference between the wild type and each mutant protein being observed.

Subsequently, we have tested, with GST pull-down assays [42], the possible *in vitro* interaction of the amino acid transceptor Gap1 with Sch9, and also with Pkh1 as control. Pkh1, considered as a homolog of mammalian PDK1, is a confirmed Sch9 upstream kinase [43,44]. HA-tagged Gap1 (HA-Gap1) was expressed in yeast on a plasmid under its own promotor. GST tag only (GST, as a negative control), GST-tagged Sch9 (GST-Sch9) and Pkh1 (GST-Phk1) were expressed in *E. coli*. Gap1–Sch9 and Gap1–Pkh1 interactions were subsequently tested separately by the pull-down assay (procedure see Materials and methods). Precipitated HA-tagged Gap1 was detected by Western blot with an anti-HA-HRP antibody. A clear signal of precipitated HA-Gap1 was observed indicating that Gap1 also binds with Sch9 *in vitro* (Figure 2A). Weaker signals were detected for interaction between HA-Gap1 and GST-Pkh1 (Figure 2A). After the construction of the appropriate BiFC strains, we could not detect any *in vivo* BiFC signal for the interaction of Pkh1 with Gap1 (Supplementary Figure S1C). This may be due to the weaker expression of the kinase in nitrogen-starved cells, their intracellular compartmentation, weaker interaction, or to limitations of the BiFC methodology [45]. Hence, our results demonstrate that there is strong interaction both *in vitro* and *in vivo* between Gap1 and Sch9, opening up the possibility for functional interaction between the two proteins, possibly regulated by nitrogen availability.

### Mapping with peptide microarray reveals a potential Gap1 domain for Sch9 interaction

**Figure 3.**
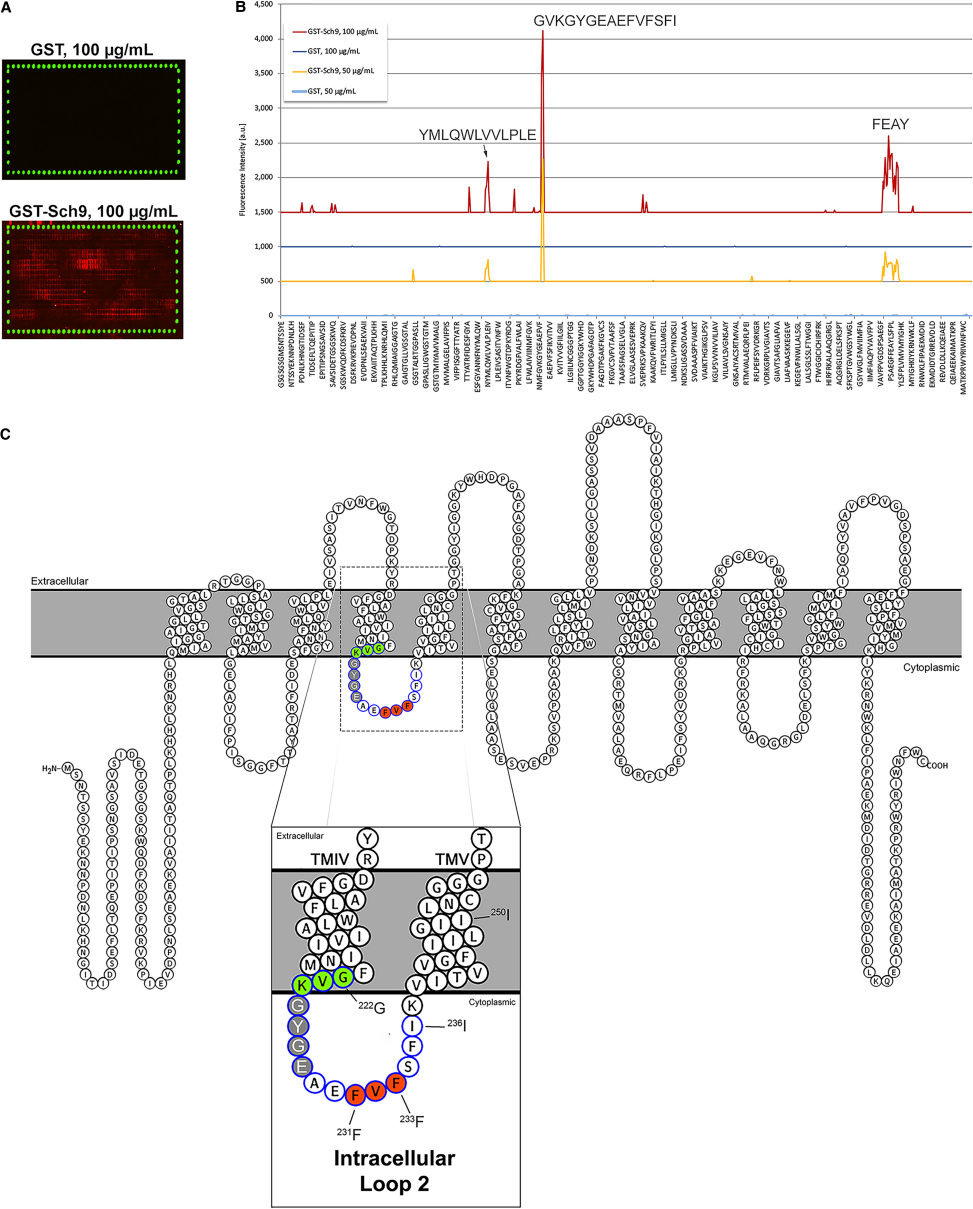
Identification of putative binding domains in Gap1 for interaction with Sch9 by mapping with a peptide microarray. A Gap1 peptide microarray was constructed and used for mapping the putative Sch9 binding site using 50 or 100 µg/ml GST-Sch9, or only GST as negative control (for description of peptide microarray, see Supplementary document S2). (**A**) Microarray scan figures of GST or GST-Sch9 at concentration of 100 µg/ml. *Upper figure*: no interaction signal is observed on the microarray incubated with GST negative control. *Lower figure*: a range of positive signals (contiguous red spots) is observed on the microarray incubated with GST-Sch9. The HA peptides are synthesized on the edge of the peptide microarray, which are tested with anti-HA (12CA5) DyLight800 antibody after the mapping, and shown as green dots in the microarray scan figure. They are used as an internal control to confirm the assay quality and to facilitate grid alignment for data quantification. The clear and complete green dots of these control peptides validate the overall peptide microarray integrity and assay quality. (**B**) Quantification of the signals from the microarray scans is shown as intensity plots. For a better comparison of the response profiles with the two concentrations of GST and GST-Sch9, the intensity plots were shifted (the lines for GST-Sch9 at 50 µg/ml, GST at 100 µg/ml and GST-Sch9 at 100 µg/ml, were up-shifted with 500, 1000 and 1500 a.u., respectively). The control assays with pure GST did not reveal any background interaction at both protein concentrations. In contrast, many weak to moderate interactions were observed with GST-Sch9 at both protein concentrations, of which the strongest interactions were labeled on top of the peaks. (**C**) Location of the putative Sch9 binding domain in the predicted Gap1 topology (generated by Protter: http://wlab.ethz.ch/protter/start/) [47]. Gap1 has 602 amino acid residues, arranged in 12 transmembrane domains (TMDs), with cytosolic N- and C-termini. The putative interaction motif ‘GVKGYGEAEFVFSFI’, indicated with circles with blue lining, is located in intracellular loop 2, with the ^231^FVF^233^ residues which are most critical for Sch9 binding and further investigated by mutagenesis, indicated with circles filled in red.

To identify putative domains in Gap1 responsible for the interaction with Sch9, mapping with Gap1 peptide microarray [46] was performed by custom order at PEPperPRINT (www.pepperprint.com). A Gap1 peptide microarray was constructed and binding of the peptides with GST-Sch9 or GST tag only as a negative control, purified from *E. coli*, was determined. For microarray details and mapping procedure, see Materials and methods. The microarray scan figures showed that no interaction was observed with only the GST tag, while a range of positive signals was observed with GST-Sch9 (red spots in Figure 3A). In the intensity plot, quantified from the microarray scan figures, the control assays with pure GST did not reveal any background interaction. In contrast, many weak to moderate interactions were observed with GST-Sch9 at both protein concentrations (Figure 3B), showing interactions with peptides containing the consensus motifs ‘^175^YMLQWLVVLPLE^186^’, ‘^531^FEAY^534^’ and particularly ‘^222^GVKGYGEAEFVFSFI^236^’, which was the strongest. The most plausible ‘^222^GVKGYGEAEFVFSFI^236^’ motif was later confirmed by an amino acid residue substitution scan (see further).

Based on the predicted Gap1 topology from Protter (http://wlab.ethz.ch/protter/), the identified ‘^222^GVKGYGEAEFVFSFI^236^’ putative binding motif localizes in intracellular loop 2 of Gap1 (Figure 3C). It has been shown previously that mutations in this loop do not affect Gap1 sorting to the plasma membrane, but influence Gap1 transport activity. The ‘^222^GVK^224^’ and ‘^225^GYGE^228^’, as indicated in Figure 3C with circles filled in light green or dark gray, that upon mutagenesis into alanines result in reduced or abolished Gap1 transport activity, respectively [48]. Blastp of the identified ‘GVKGYGEAEFVFSFI’ motif against *S. cerevisiae* reference proteins was performed at NCBI, which did not reveal any significant sequence similarity with the other identified Sch9-associated transceptors. This indicates that the other transceptors may bind Sch9 through different domains compared with Gap1. On the other hand, conservation of this domain was observed among multiple amino acid permeases, according to the protein sequence alignment (Supplementary Figure S3B). As negative controls for the Gap1–Sch9 BiFC interaction, the interaction of Sch9 with the high-affinity glutamine permease Gnp1 and low-affinity methionine permease Mup3 was tested by constructing Gnp1–Sch9 and Mup3–Sch9 BiFC strains. Gnp1 has less sequence similarity in the tail part of the Sch9-binding motif (which was found to be important for the binding in a substitution scan, as described below), while Mup3 has no sequence similarity with the domain at all. In both cases, no BiFC interaction was observed, both in exponential growing cells and in 24 h nitrogen-starved cells (Supplementary Figure S3C). This indicates that Sch9 does not simply bind to any amino acid or ammonium transporter, but rather that a specific sequence in the binding domain is important.

**Figure 4.**
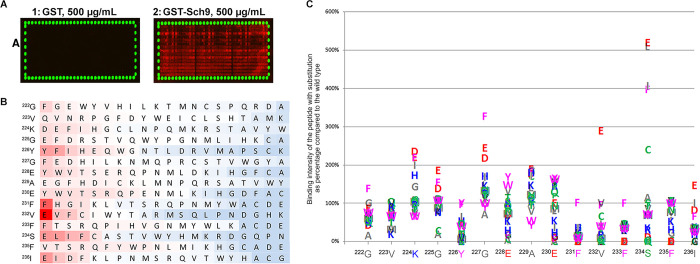
Substitution scan of the Gap1 peptide ^222^GVKGYGEAEFVFSFI^236^ for Sch9 binding. A substitution peptide microarray was constructed and screened for binding with 100 or 500 µg/ml GST-Sch9, or only GST as negative control (for description of peptide microarray, see Supplementary document S2). (**A**) Microarray scans of GST or GST-Sch9 at a concentration of 500 µg/ml. *Scan* Figure 1: no interaction is observed on the substitution microarray incubated with GST negative control. *Scan* Figure 2: a range of positive signals (contiguous red spots) is observed on the substitution microarray incubated with GST-Sch9. The HA peptides were synthesized on the edge of the peptide microarray, which are tested with anti-HA (12CA5) DyLight800 antibody after the mapping, and shown as green dots in the microarray scan figure. They are used as an internal control to confirm the assay quality and to facilitate grid alignment for data quantification. The clear and complete green dots of these control peptides validate the overall peptide microarray integrity and assay quality. (**B**) The substitution matrix highlights the binding with GST-Sch9 for any given amino acid residue substituted by all 20 amino acid residues using color coding (red for stronger binding, blue for weaker binding) and re-arranged according to the strength of GST-Sch9 binding. The color intensity represents binding intensity with GST-Sch9 calculated by dividing the spot intensity for a given modified peptide by the average spot intensity for all 20 peptides substituted at the same amino acid position. (**C**) The amino acid plot shows GST-Sch9 binding with the different modified peptides for each amino acid residue at a given position. The binding intensity was calculated by dividing the spot intensity of a given peptide by the spot intensity of the wild type peptide and is shown as percentage of binding with the wild type peptide.

To further confirm the relevance of the potential binding motif described above and to determine the importance of each amino acid residue in the motif (‘^222^GVKGYGEAEFVFSFI^236^’), an amino acid substitution scan was performed by PEPperPRINT. In the substitution scan, individual amino acid residues at each position of the ‘^222^GVKGYGEAEFVFSFI^236^’ peptide were replaced by all 20 amino acids. Subsequently, the interaction between each modified peptide and GST-Sch9 was determined. The microarray scan figures showed that the GST tag alone, used as negative control, did not reveal any background binding reaction. Replacement of residues at different positions in the peptide led to an alteration in binding intensity with GST-Sch9 (Figure 4A). The intensity plot quantified from the microarray scan figure showed reduced or enhanced binding intensity among the substituted and the wild type peptides (see raw data in Supplementary document S4). This confirmed the results from the peptide mapping, as the exchange of amino acid residues caused changes in the GST-Sch9-binding intensity, which were apparently not due to changes in the hydrophobic character of many residues in the domain.

The substitution matrix, in which binding intensity was plotted against the average spot intensity for all 20 peptides substituted at the same amino acid position (Figure 4B) and the amino acid plot, in which binding intensity was plotted against the spot intensity of the wild type peptide (Figure 4C), highlighted a critical motif ‘^231^FVFSFI^236^’ and a variable stretch ‘^222^GVKGYGEAE^230^’. The ^231^F, ^233^F and ^232^V turned to be the most important residues for the binding, as ^231^F and ^233^F did not tolerate substitution by any other amino acid without a loss in the binding of at least 50%. ^232^V could be exchanged by F or V without significant effect on Sch9 binding, while the replacement by E even resulted in a 3-fold increase in spot intensity. For amino acid residue ^234^S, exchange into E, L, I, F or C caused a significant increase in Sch9 binding. The ^235^F and ^236^I were less important but still showed dramatically reduced binding upon replacement with multiple residues. All the residues in this domain could tolerate some replacements and maintain the binding with GST-Gcd6.

### Effect of mutations in the putative Gap1–Sch9-binding site on *in vitro* binding of Gap1 with Sch9 and on Gap1-dependent upstart of cell proliferation

We constructed Gap1^F231D^, Gap1^V232K^ and Gap1^F233D^ mutant forms of Gap1 in the identified putative Sch9 binding site. They were expressed under the control of the *GAP1* promotor using the single-copy pFL38 vector. The three amino acid residues are located in the motif ^231^FVFSFI^236^ and were the most critical for binding with GST-Sch9 in the substitution scan results, showing zero spot intensity in binding. We also constructed a mutant form Gap1^FVF-DKD^ that combines the three mutations above, and Gap1^S234E^ which showed more than 5-fold higher spot intensity compared with the wild type peptide (Figure 4C).

The Gap1 deletion strain (JT20867) was transformed with empty plasmid or plasmids encoding HA-tagged wild type (Gap1^WT^) or one of the Gap1 mutant forms (Gap1^FVF-DKD^, Gap1^F231D^, Gap1^V232K^, Gap1^F233D^ and Gap1^S234E^, as described above). Physical interaction between Sch9 and these mutant forms of Gap1 was evaluated with GST pull-down assays. The Western blot band intensity was quantified and calculated by Fiji (see Materials and methods). However, no significant difference was observed between the wild type and mutant forms of Gap1 for binding with GST-Sch9 (Figure 2B), which is different from what we observed in the microarray mapping with the isolated peptides. There are several possible explanations. First of all, the binding with isolated peptides may be different from that with the native protein, because the peptides may be tucked inside the protein or because their conformation in the native protein may be different from that of the isolated peptide. In addition, there may be multiple interaction sites between the native, full-length Gap1 and the Sch9 protein so that mutagenesis of a single interaction site is not enough to abolish the interaction completely.

**Figure 5.**
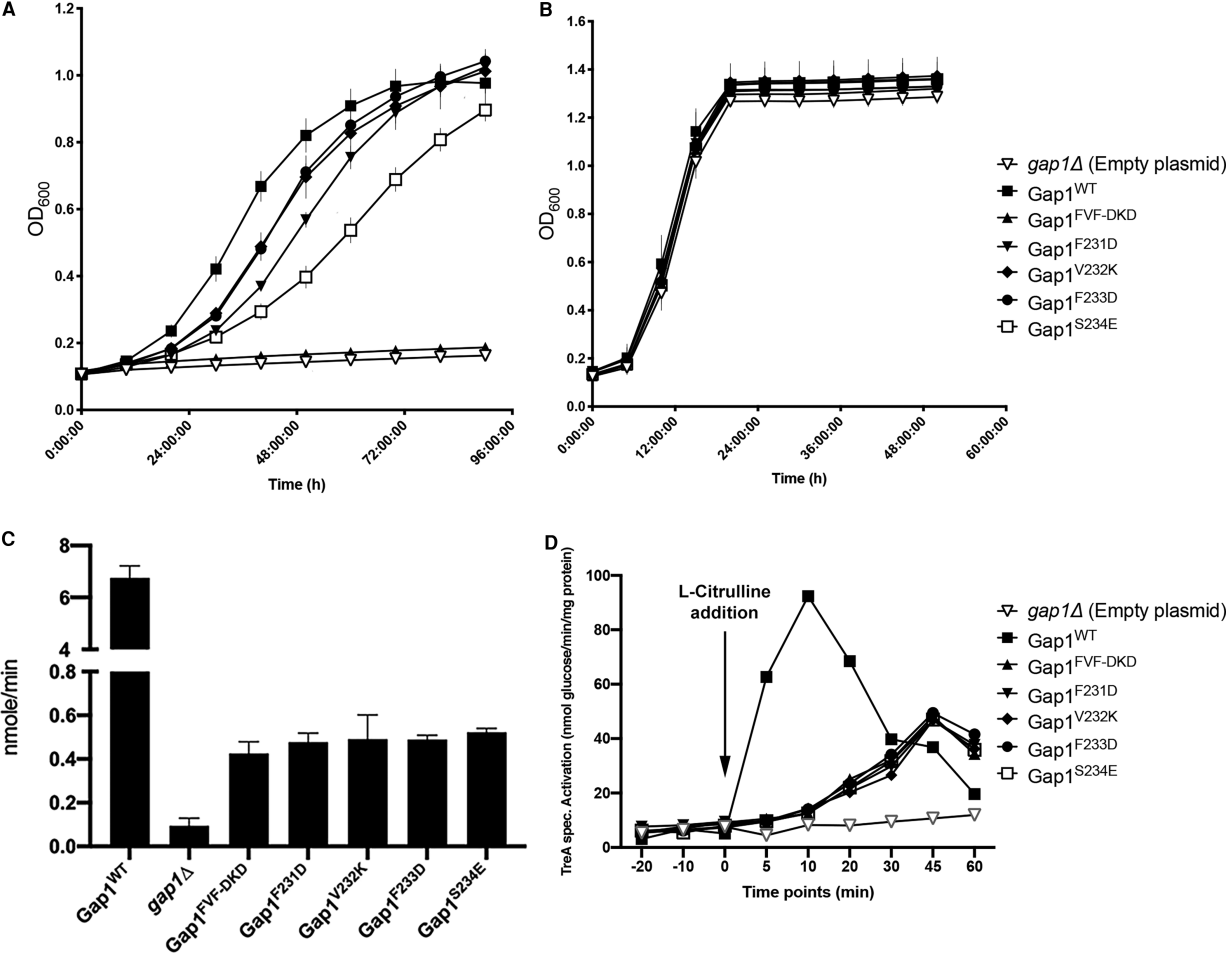
Re-upstart of growth in nitrogen-starved cells expressing wild type or Gap1 forms containing mutations in the putative Sch9 interaction domain. (**A**) Re-upstart of growth in NSM with 1 mM L-citrulline. OD_600nm_ was measured every 1 h for 90 h. (**B**) Re-upstart of growth in SC-URA medium. OD_600nm_ was measured every 1 h for 50 h. Standard deviation of the average of four replicates is shown with the error bars (for more details, see Materials and methods). (**C**) Transport of 1 mM [^3^H]-labeled L-citrulline was measured for 1 min. Standard deviation of the average of three replicates is shown with the error bars. (**D**) As a read-out for Gap1-mediated PKA activation, short-term trehalase activation by the Gap1 mutant forms was measured. 5 mM L-citrulline was added after 24 h nitrogen starvation at time point 0. Trehalase activity was measured at different time points. Strains: *gap1Δ* strain transformed with empty plasmid (▽), plasmid expressing Gap1^WT^ (▪), Gap1^FVF-DKD^ (▴), Gap1^F231D^ (▾), Gap1^V232K^ (◆), Gap1^F233D^ (●) or Gap1^S234E^ (□).

Subsequently, the effect of the Gap1 mutant forms on cell growth was evaluated by measuring the re-upstart of growth in nitrogen-starved cells upon re-addition of L-citrulline. Cells starved 24 h for nitrogen, which display high expression of Gap1 at the plasma membrane [49], were inoculated either in NSM with 1 mM L-citrulline as nitrogen source, or in SC-URA medium, which contains ammonium and all amino acids. Low concentrations of L-citrulline (e.g. 1 mM) are exclusively taken up by Gap1 [6,50]. The upstart of growth in NSM with 1 mM L-citrulline was retarded to different extents for all strains with a Gap1 mutant form (Figure 5A). The strains with Gap1^V232K^ or Gap1^F233D^ showed similarly weak retardation compared with the strain with Gap1^WT^. The strains with Gap1^F231D^ or Gap1^S234E^ showed stronger retardation, while the strain with Gap1^FVF-DKD^ did not start to grow at all, just like the *gap1Δ* strain transformed with empty plasmid. On the other hand, in SC-URA medium all strains showed the same rapid upstart of growth like the strain with Gap1^WT^ (Figure 5B). In this medium, the upstart of growth is not dependent on Gap1 since ammonium and amino acids can be taken up by alternative transporters [51]. Hence, in spite of the apparent absence of a loss in binding affinity between Gap1 and Sch9, at least when evaluated with *in vitro* pull-down assays, the specific mutations engineered in Gap1 based on the results of the peptide mapping experiments, caused clear negative effects on Gap1-dependent growth. Uptake activity of 1 mM [^3^H]-labeled L-citrulline was also measured with a 1min assay to evaluate the transport capacity of the Gap1 mutant forms (Figure 5C). Surprisingly, all the mutant forms showed similar uptake efficiency which was less than 10% of the wild type Gap1. The PKA signaling capacity of the mutant Gap1 forms was also tested by measuring L-citrulline-induced trehalase activation, which showed that the Gap1 mutant forms displayed an impaired signaling capacity correlated with the reduction in their transport capacity (Figure 5D). Apparently, the transport and PKA signaling capacities of Gap1 do not correlate with the rate of cell growth. These results indicate that the Gap1 mutant forms must regulate the re-upstart of growth in a way unrelated to amino acid uptake and PKA signaling and thus apparently through one or more other Gap1-dependent regulatory controls, for instance by interaction with Sch9 and/or other targets.

## Discussion

The discovery of nutrient transceptors that control a major signaling pathway with pervasive effects on cell physiology and development like the PKA pathway in yeast raises the question whether nutrient transceptors may interact with other major regulatory proteins to control cellular properties, similar to the widespread effects of classical receptors on cellular metabolism and behavior. Nutrient transceptors are actually likely candidates for the evolutionary precursors of classical receptors in higher multicellular organisms [52]. In this paper, we provide evidence that Gap1 interacts with the Sch9 protein kinase suggesting that transceptors may play hitherto unrecognized roles in nutrient sensing for the regulation of cellular properties.

The TOR pathway and PKA have been suggested to function in parallel for nutrient sensing and cell growth [53,54]. Moreover, they share common substrates like Rim15 [55] and Flo11 [56]. Apparently, the protein kinase Sch9 is involved in both the TOR and the PKA pathways in response to nutrient repletion [57]. On one hand, Sch9 is directly phosphorylated by TORC1 and as such commonly used as a read-out for TORC1 activation [17]. On the other hand, Sch9 is also important for nitrogen-induced activation of the PKA pathway, as its deletion abolishes Gap1 signaling to PKA [24]. However, this does not necessarily imply that Sch9 is an intermediate in the signaling pathway from Gap1 to PKA. Sch9 could also be required for the proper functioning of one of the components of the signaling pathway or even affect Gap1 or PKA functionality itself. Hence, our results do not imply that the putative Gap1–Sch9 amino acid signaling is involved in Gap1-mediated activation of PKA. The two systems may act in parallel. If Gap1–Sch9 interaction is involved in amino acid-induced upstart of growth, our results would actually contradict this possibility, since amino acid transport activity of Gap1 is clearly correlated with amino acid activation of the PKA target trehalase but not with the rate of amino acid-induced upstart of growth in nitrogen-starved cells. Interaction between the TOR and the PKA pathways is supposed to happen through Sch9, since it has been reported that TOR regulates the phosphorylation of the PKA regulatory subunit Bcy1 through Sch9 [58,59]. On the other hand, the TORC1 pathway does not appear to be involved in nutrient transceptor-mediated short-term activation of the PKA pathway in nutrient-starved cells [54].

Sch9 is a well-established phosphorylation target of TORC1, which suggests that nutrient regulation of Sch9 is exerted by the nutrient-sensing systems that have been suggested to control TORC1 activity. However, the latter have been particularly difficult to identify and also seem to be much more specific than previously anticipated. In yeast, the molecular connection between nutrient availability and TORC1 has only been shown for leucine with the involvement of leucyl-tRNA synthetase Cdc60 [60,61]. Although a correlation between the quality of the nitrogen source and the requirement for glutamine biosynthesis for activation of TORC1, based on Sch9 phosphorylation as read-out, has been documented, the molecular mechanism connecting glutamine biosynthesis to TORC1 has remained unclear [62]. Also in mammalian cells, identification of amino acid sensing proteins for activation of mTORC1 is limited until now to sensing of leucine and arginine [63]. The transceptor–Sch9 interaction identified in this work may provide a mechanism for regulation of Sch9 by multiple nutrients, since transceptors have been discovered for amino acids, ammonium, phosphate, sulfate and even for the micronutrients iron and zinc. Moreover, it cannot be excluded that transceptors also exist for regulation by other nutrients, like sugars, in connection with or independent of GPCR — cAMP regulation of the PKA pathway.

Our *in vivo* and *in vitro* interaction results link nutrient transceptors to Sch9. Apparently, like regular receptors, nutrient transceptors can directly interact with major downstream regulatory proteins. This offers an alternative explanation for nutrient control of Sch9 phosphorylation parallel with TORC1. Sensing of the nutrient substrate by the transceptor may modulate the conformation of Sch9 rendering Sch9 available for (increased) phosphorylation by the TORC1 protein kinase. TORC1 is known to be responsible for sensing intracellular nutrients [64], while nutrient transceptors are involved in sensing of extracellular nutrients [7,8]. Sch9 may be a focal point where the extra and intracellular nutrient signals are integrated to provide a common response.

While the Mep2, Sul1 and Pho84 BiFC complexes with Sch9 were at least to a significant extent present at their expected plasma membrane location in the cell, the Gap1–Sch9 BiFC complex was entirely located at the NV junction. Since L-citrulline uptake was only reduced with about 15% in the Gap1–Sch9 BiFC cells, Gap1 must still be located abundantly in the plasma membrane. Moreover, Gap1 must be present in large excess in the cell compared with Sch9, since no fluorescence was detectable at the level of the plasma membrane. This difference in expression level is consistent with the house-keeping and regulatory function of Gap1 and Sch9, respectively. Apparently, no Gap1–Sch9 BiFC complex could reach the plasma membrane. The most likely explanation appears to be that the tight, persistent binding between the two half-citrine fusion proteins creates a large, rigid Gap1–Sch9 complex that disturbs intracellular sorting of the complexed Gap1 in such a way that it ends up at the NV junction. For some reason, the other transceptor–Sch9 BiFC complexes do not suffer from this problem and are sorted properly to the plasma membrane and apparently also to the vacuole in the case of Sul1 and Pho84.

TORC1 and its regulators in yeast have been known to localize to the vacuolar membrane [65]. The phosphorylation of Sch9 by TORC1 requires vacuolar localization of Sch9, which is mediated by the PI(3,5)P2-binding C2 domain of Sch9 [66,67]. It has also been demonstrated that under oxidative stress, Sch9 is released from the vacuole membrane for inhibition [66]. The observed Gap1–Sch9 BiFC interaction signal colocalizes with Nvj1-RFP at the NV junction, to which part of the perinuclear ER also attaches [28,68]. PMN at the NV junction is a selective autophagic process in yeast cells that targets parts of the nucleus for degradation. PMN can be induced by nitrogen starvation or rapamycin addition, and is regulated by the rapamycin-sensitive TORC1 complex [69]. It is also well known that nitrogen starvation causes TORC1 inactivation, which leads to dephosphorylation and hence inactivation of Sch9 [70]. Based on these literature data and our observation that the Gap1–Sch9 BiFC complex is located at the NV junction, an alternative hypothesis can be proposed. Under nitrogen starvation conditions, the ER-localized Gap1 could recruit vacuole membrane-localized Sch9 to the NV junction through the Gap1–Sch9 interaction. This translocation of Sch9 further inhibits its activity by separating it from TORC1 or even by sending Sch9 to the vacuole for degradation through the PMN. The suggestion that Sch9 has less affinity to TORC1 than other TORC1 substrates may support this hypothesis [71]. The binding of free Sch9 by the other transceptors at the plasma membrane may also be responsible for the recruitment of free Sch9, possibly with a different mechanism compared with Gap1.

The use of peptide microarrays has been a rapidly growing technology with a broad range of applications. It has been developed for almost 30 years and commercialized for the past decade, with multiple mature methodologies now available [46]. Peptide microarrays normally consist of hundreds to thousands distinct but overlapping peptides with an appropriate number of replicates. They have been successfully used in the search for epitopes for antibody binding [72,73], protein-peptide binding studies [74], cell-adhesive peptide identification [75], etc. On the other hand, their largest disadvantage is that the peptides hardly retain the conformation they had in the original protein [46]. Using the Gap1 peptide and substitution peptide microarrays, synthesized by PEPperPRINT, we identified Gap1 peptides binding with Sch9 and also identified the most critical amino acid residues in the peptides for the interaction with Sch9. These peptides and amino acid residues suggest, but do not predict with certainty, the Gap1 binding domain for the Gap1–Sch9 interaction *in vivo*. Mutagenesis, however, of the most critical residues identified in this way did not result in altered binding in the *in vitro* GST pull-down assays, as quantified from the Western blot bands. This may be due to the fact that the free peptides do not have the same conformation as the integrated peptides in the native protein, to the presence of multiple binding sites between Sch9 and Gap1 or to the fact that the *in vitro* conditions do not reflect properly the intracellular conditions for Gap1–Sch9 binding. The BiFC interaction, which takes place in the natural *in vivo* intracellular environment could at least to some extent overcome these problems. Hence, a detailed study of single and multiple Gap1 mutations, engineered by CRISPR/Cas9 technology [76–78], on the binding between Gap1 and Sch9, as detected *in vivo* by BiFC, could finally lead to mutant forms with absent binding. These would be very useful tools to study the physiological consequences on different readouts known to be affected by Gap1 or Sch9. On the other hand, mutagenesis of the most critical residues identified with peptide mapping already affected Gap1 transport activity, which indicates that introduction of multiple mutations to abolish Gap1–Sch9 binding *in vivo* completely without compromising transport activity, might be highly challenging. On the other hand, the mutagenesis of single residues also produced already clear negative effects on Gap1-dependent re-upstart of growth. This could have simply been due to reduction in Gap1 amino acid uptake activity. However, although the different mutations did reduce L-citrulline uptake activity in short-term uptake assays, which was correlated with reduced activation of the PKA target, trehalase, there was no correlation with the retardation of the upstart of Gap1-dependent growth over the next 90 h. Hence, the mutations in Gap1 may affect a functional interaction between Gap1 and Sch9 rather than a structural interaction. Aberrant functional interaction between Gap1 and Sch9 may thus have been responsible for the compromised re-upstart of growth in the strains with different mutant Gap1 forms.

A particular phenomenon in which transceptor–Sch9 interaction could play a major role, is cellular aging. Calorie restriction and nutrient limitation are well-known to increase longevity in many organisms [79]. These are precisely the conditions in which the nutrient transceptors that interact with Sch9 are strongly up-regulated. Inactivation of Sch9/PKB is well known to increase longevity in yeast and higher eukaryotic organisms [80]. Hence, down-regulation of Sch9 by nutrient transceptor interaction in nutrient-limited cells could (in part) be responsible for the positive effect of nutrient starvation on longevity. One of the proposed models for the positive effect of nutrient limitation on longevity is that Tor1, Sch9 and Ras/PKA deficiency all lead to more functional Rim15 kinase, as well as its downstream effectors, Msn2/4 and Gis1. The improved cellular protection provided by the up-regulated stress response is thought to be the key for establishing superior longevity [81,82]. Cell longevity in nitrogen starvation conditions could be determined to evaluate the possible involvement of the Gap1–Sch9 interaction in the control of longevity. However, besides Gap1, other transceptors and multiple putative transceptors are also induced during nitrogen starvation. For instance, the ammonium transceptor, Mep2, is known to be strongly induced during nitrogen starvation and our current work has shown that it also binds with Sch9. Hence, Mep2 may be responsible together with Gap1 and other nitrogen starvation-induced transceptors for sequestrating Sch9 and in this way increase longevity. Such a mechanism, in which multiple nutrient-sensing proteins control a single regulatory target protein would be difficult to identify because the redundant sensors could compensate for each other.

## Conclusions

Our work has revealed that yeast nutrient transceptors physically interact with Sch9, a major regulatory protein kinase involved in multiple nutrient-controlled processes in yeast. Mutations engineered in a putative Gap1 transceptor — Sch9 interaction site affect transceptor-dependent upstart of growth in a way that is not correlated with reduction in amino acid uptake. Hence, our results suggest that nutrient transceptors may play a role in nutrient regulation of Sch9 activity and its function in the control of cell growth, and by extension that nutrient transceptors may regulate major cellular properties by interaction with regulatory protein kinases.

## Abbreviations

BiFC: bimolecular fluorescence complementation
PKA: protein kinase A
TOR: target of rapamycin
ER: endoplasmic reticulum
PMN: piecemeal microautophagy of the nucleus
NV: nucleus–vacuole
DIC: differential interference contrast
