# Supplementary material for: Nutrient transceptors physically interact with the yeast S6/protein kinase B homolog, Sch9, a TOR kinase target
Source: Biochem J. 2021 Jan 27;478(2):357–75. doi: 10.1042/BCJ20200722 (PMC7850899; doi:10.1042/BCJ20200722)
Supplement: Supplementary Figures S1-S3 [file BCJ-478-357-s1.pdf]

Supplementary figure legends

Figure S1. Negative controls for BiFC. A. No BiFC signal was observed in cells of strains for determination of *in vivo* Gap1-Sch9, Mep2-Sch9, Sul1-Sch9 and Pho84-Sch9 interaction. Cells were cultivated in SC medium to exponential phase and directly loaded on a slide for microscopy observation. B. Absence of a BiFC signal in cells of strains with split-citrine single-tagged Sch9, Gap1, Mep2, Sul1 or Pho84 (N-citrine, tagged with citrine N-terminus half; C-citrine, tagged with citrine C-terminus half). Cells were starved for the specific nutrient transceptor substrate and in the case of Sch9-C-citrine starved for nitrogen. The cells were directly loaded on a slide for microscopy observation. C. Absence of a detectable BiFC signal in the Gap1-Pkh1 strain (Gap1-N-citrine/Pkh1-C-citrine). Cells were starved for nitrogen for 24h and directly loaded on a slide for microscopy observation. Images for the BiFC citrine fluorescent signal (left) and the DIC chanel (right) are shown. All the scale bars indicate 10µm.

Figure S2. Gap1-Sch9 BiFC signal locates at the nucleus-vacuole junction. The cells were starved for nitrogen for 24h before staining or visualization in the confocal microscope. For the staining procedures, see Materials and Methods. The first column shows the Gap1-Sch9 BiFC citrine fluorescent signal, the second column shows the marker signals for different organelles, the third column shows the merged fluorescence signals of the first two columns and the last column shows the DIC pictures. The dyes or proteins used as organelle markers are indicated in front of the rows. The nucleus, stained by DAPI in blue, always showed attachment of the BiFC signal. The ER marker Kar2-mCherry showed partial colocalization with the BiFC signal in its perinuclear part. The DsRed tagged Golgi specific marker protein Sec7 did not show any colocalization with the BiFC signal. Vacuolar membrane staining with FM4-64 showed partial colocalization with the BiFC signal in its perinuclear part. Perfect colocalization

between the BiFC signal and Nvj1-RFP was observed, with the shape of the stripes or arcs a bit more extended, probably because of the double fluorescent tagging. All the scale bars indicate 10µm.

Figure S3. A. The uptake activity of 1mM [^{3}H] labeled L-citrulline was measured in BY prototrophic strain and the Gap1-Sch9 BiFC strain. 1min L-citrulline uptake by 24h nitrogen-starved cells. A significant difference between the two strains was shown by a t-test (P=0.0006). Standard deviation of the average of three replicates is shown with the error bars. B. After a BLAST search with the binding domain, the sequence of the area encompassing the binding domain was aligned in the 9 most closely related amino acid permeases with the corresponding sequence in Gap1 using Clustal Omega [1]. Multiple permeases showed high conservation with the domain identified in Gap1. Residues which are identical among >80% of all the sequences are marked in dark grey. Residues which are similar among >80% of all the sequences are marked in light grey. C. Gnp1-Sch9 and Mup3-Sch9 BiFC cells did not show any BiFC signal neither in exponential phase nor in stationary phase after 24h nitrogen starvation. Images of BiFC citrine fluorescent signal (left) and the DIC chanel (right) are shown. All the scale bars indicate 10µm.

Reference

1 Madeira, F., Park, Y. M., Lee, J., Buso, N., Gur, T., Madhusoodanan, N., Basutkar, P., Tivey, A. R. N., Potter, S. C., Finn, R. D. and Lopez, R. (2019) The EMBL-EBI search and sequence analysis tools APIs in 2019. Nucleic Acids Res. **47**, W636-W641 https://doi.org/10.1093/nar/gkz268

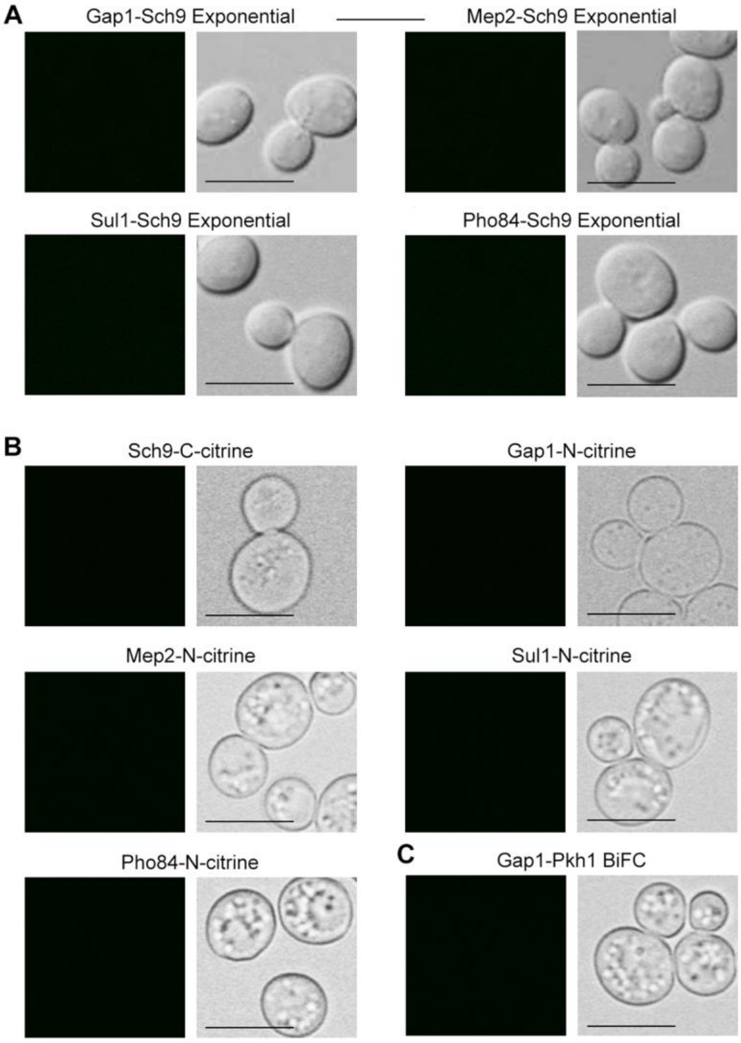
A
Gap1-Sch9 Exponential
Mep2-Sch9 Exponential
Sul1-Sch9 Exponential
Pho84-Sch9 Exponential
B
Sch9-C-citrine
Gap1-N-citrine
Mep2-N-citrine
Sul1-N-citrine
Pho84-N-citrine
C
Gap1-Pkh1 BiFC

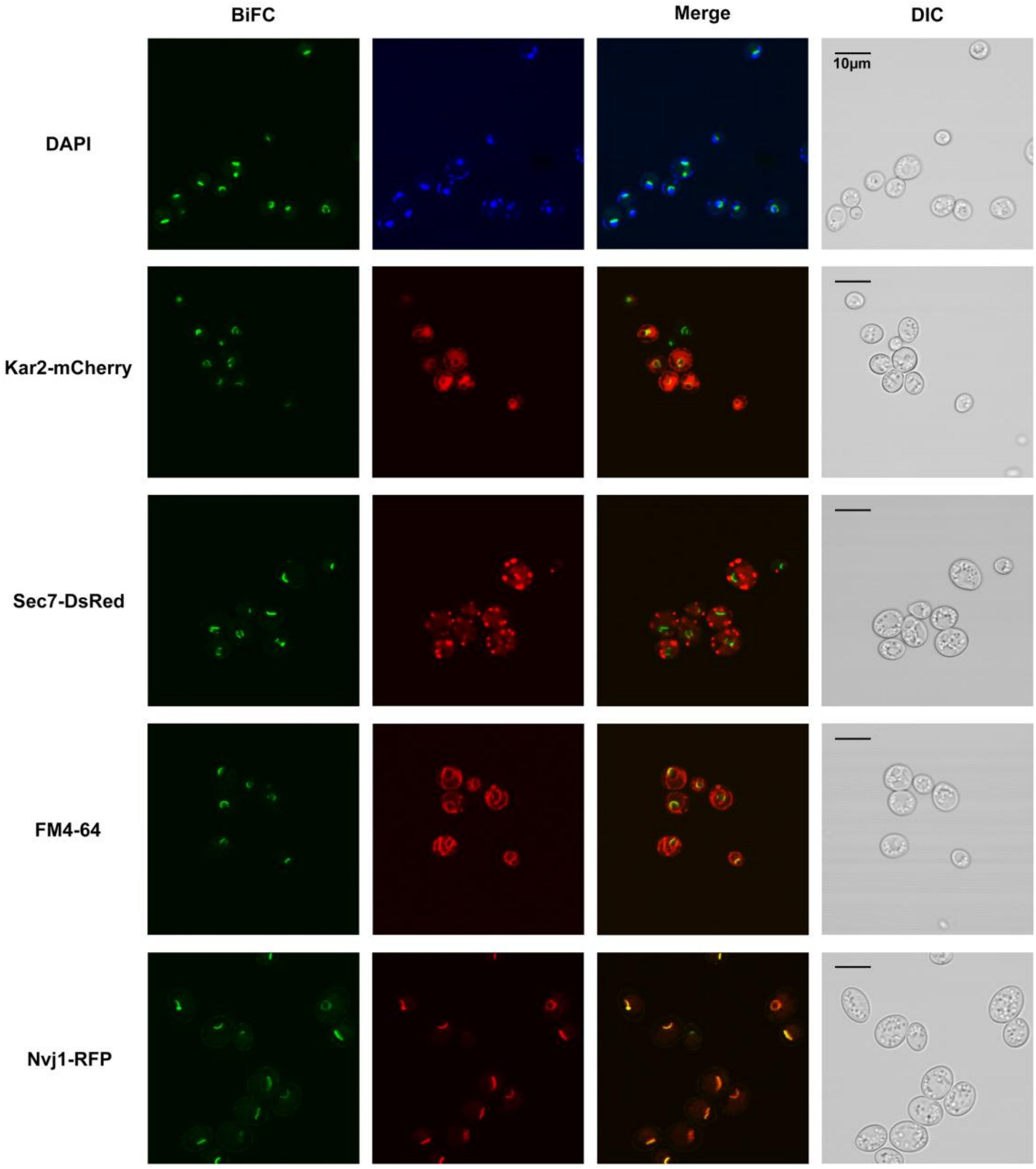
BiFC
Merge
DIC
DAPI
Kar2-mCherry
Sec7-DsRed
FM4-64
Nvj1-RFP
10μm

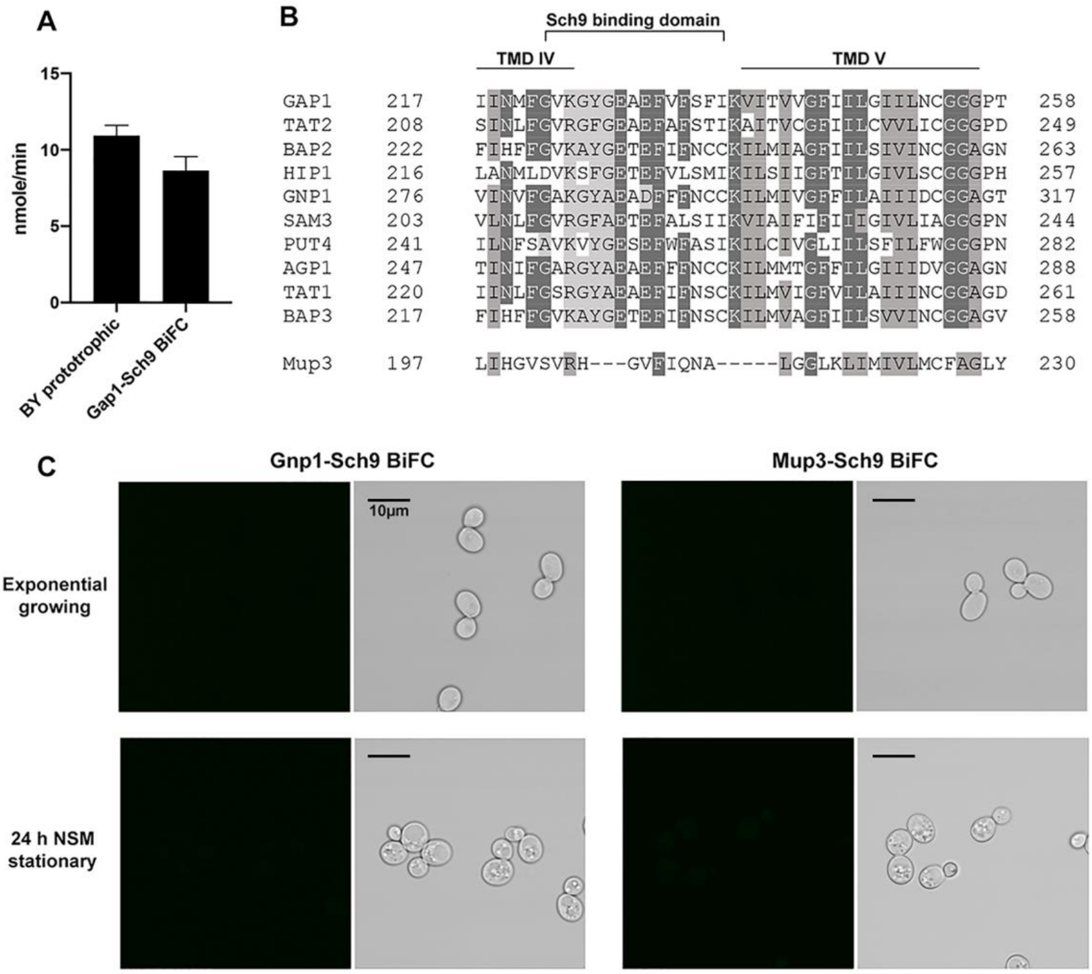
A
15
10
5
0
nmole/min
BY prototrophic
Gap1-Sch9 BiFC
B
Sch9 binding domain
TMD IV
TMD V
GAP1 217 IINMFGVKGYGEAEFVFSFIKVITVVGFIILGIILNCGGGPT 258
TAT2 208 SINLFGVRGFGEAEFAFSTIKAITVCGFIILCVVLICGGGPD 249
BAP2 222 FIHFFGVKAYGETEFIFNCCKILMIAGFIILSIVINCGGAGN 263
HIP1 216 LANMLDVKSFGETEFVLSMIKILSIIGFTILGIVLSCGGGPH 257
GNP1 276 VINVFGAKGYAEADFFFNCCKILMIVGFFILAIIIDCGGAGT 317
SAM3 203 VLNLFGVRGFAETEFALSIIKVIAIFIFIIIGIVLIAGGGPN 244
PUT4 241 ILNFSAVKVYGESEFWFASIKILCIVGLIIILSFILFWGGGPN 282
AGP1 247 TINIFGARGYAEAEFFFNCCKILMMTGFFILGIIIDVGGAGN 288
TAT1 220 IINLFGSRGYAEAEFIFNSCKILMVIGFVILAIIINCGGAGD 261
BAP3 217 FIHFFGVKAYGETEFIFNSCKILMVAGFIILSVVINCGGAGV 258
Mup3 197 LIHGVSVRH---GVFIQNA-----LGGLKLIMIVLMCFAGLY 230
C
Gnp1-Sch9 BiFC
Mup3-Sch9 BiFC
10μm
Exponential growing
24 h NSM stationary
